# Potential roles of sex-linked differences in obesity and cancer immunotherapy: revisiting the obesity paradox

**DOI:** 10.1038/s44324-024-00007-4

**Published:** 2024-05-23

**Authors:** Logan V. Vick, Spencer Rosario, Jonathan W. Riess, Robert J. Canter, Sarbajit Mukherjee, Arta M. Monjazeb, William J. Murphy

**Affiliations:** 1 Department of Dermatology, University of California Davis School of Medicine, Sacramento, CA USA; 2 Department of Biostatistics and Bioinformatics, Roswell Park Comprehensive Cancer Center, Buffalo, NY USA; 3 Pharmacology and Therapeutics, Roswell Park Comprehensive Cancer Center, Buffalo, NY USA; 4 Department of Medicine, Division of Hematology/Oncology, UC Davis Comprehensive Cancer Center, Sacramento, CA USA; 5 Department of Surgery, Division of Surgical Oncology, University of California Davis Comprehensive Cancer Center, University of California Davis School of Medicine, Sacramento, CA USA; 6 Department of Medicine, Roswell Park Comprehensive Cancer Center, Buffalo, NY USA; 7 Department of Radiation Oncology, University of California Davis Comprehensive Cancer Center, University of California School of Medicine, Sacramento, CA USA; 8 Department of Internal Medicine, Division of Malignant Hematology, Cellular Therapy and Transplantation, University of California Davis School of Medicine, Sacramento, CA USA

**Keywords:** Cancer, Endocrinology, Oncology

## Abstract

Obesity, a condition of excess adiposity usually defined by a BMI > 30, can have profound effects on both metabolism and immunity, connecting the condition with a broad range of diseases, including cancer and negative outcomes. Obesity and cancer have been associated with increased incidence, progression, and poorer outcomes of multiple cancer types in part due to the pro-inflammatory state that arises. Surprisingly, obesity has also recently been demonstrated in both preclinical models and clinical outcomes to be associated with improved response to immune checkpoint inhibition (ICI). These observations have laid the foundation for what has been termed the “obesity paradox”. The mechanisms underlying these augmented immunotherapy responses are still unclear given the pleiotropic effects obesity exerts on cells and tissues. Other important variables such as age and sex are being examined as further affecting the obesity effect. Sex-linked factors exert significant influences on obesity biology, metabolism as well as differential effects of different immune cell-types. Age can be another confounding factor contributing to the effects on both sex-linked changes, immune status, and obesity. This review aims to revisit the current body of literature describing the immune and metabolic changes mediated by obesity, the role of obesity on cancer immunotherapy, and to highlight questions on how sex-linked differences may influence obesity and immunotherapy outcome.

## Introduction

Obesity is a condition of excess body fat that is associated with several adverse conditions, including diabetes, cardiovascular disease, and cancer^1^. The standardized metric for defining obesity, as set by the world health organization, utilizes body mass index (BMI), a value calculated using a ratio of an individual’s weight to height (weight (kg) / [height (m)]^2^). Using this metric, a BMI ≥ 30 is classified as obese, though there are exceptions based on patient demographic^3^^,^. The prevalence of obesity is rising globally, with studies indicating that rates of obesity more than tripled from 1980 to 2019^4^. In the United States, estimates have indicated that nearly 70% of adults in the US are at least overweight, with roughly 35% being classified as obese^5^. BMI is the predominant measurement for obesity, as it is easily assessed and highly predictive of adiposity, although its use as the sole diagnostic tool has been more recently considered inadequate, as BMI fails to account for important factors including body composition, adipose tissue distribution, metabolic perturbations, and ratios of lean body mass^6^. Other methodologies, such as waist, hip and thigh measurements, computed tomography (CT) or magnetic resonance imaging (MRI), have been used to account for some of these limitations, yet how to define obesity is still under debate; considering the role of diet and metabolic alterations in obesity as well as effects preclinically in different disease models, it suggests adiposity alone may not be sufficient^7,8^.

Obesity has been identified as a risk factor for at least thirteen different cancer types^9^, with studies demonstrating that obesity associated cancers account for approximately 40% of cancer diagnosis in the United States^10^. Moreover, incidence of obesity associated cancers is rising and it is predicted that obesity will surpass tobacco use as the number one preventable cause of cancer^11^. Obesity induced meta-inflammation, a condition of chronic low-grade inflammation and immune suppression, has been described as a prominent factor in increased cancer incidence, although the nutrient-rich obese environment likely also plays a role^12^. Some of the characteristics of obesity (e.g. inflammation, nutrient-rich environment) can also promote immune responses and have prompted the question as to whether there was a means to exploit these aspects of obesity and overcome the tumor-promoting effects.

**Fig. 1:**
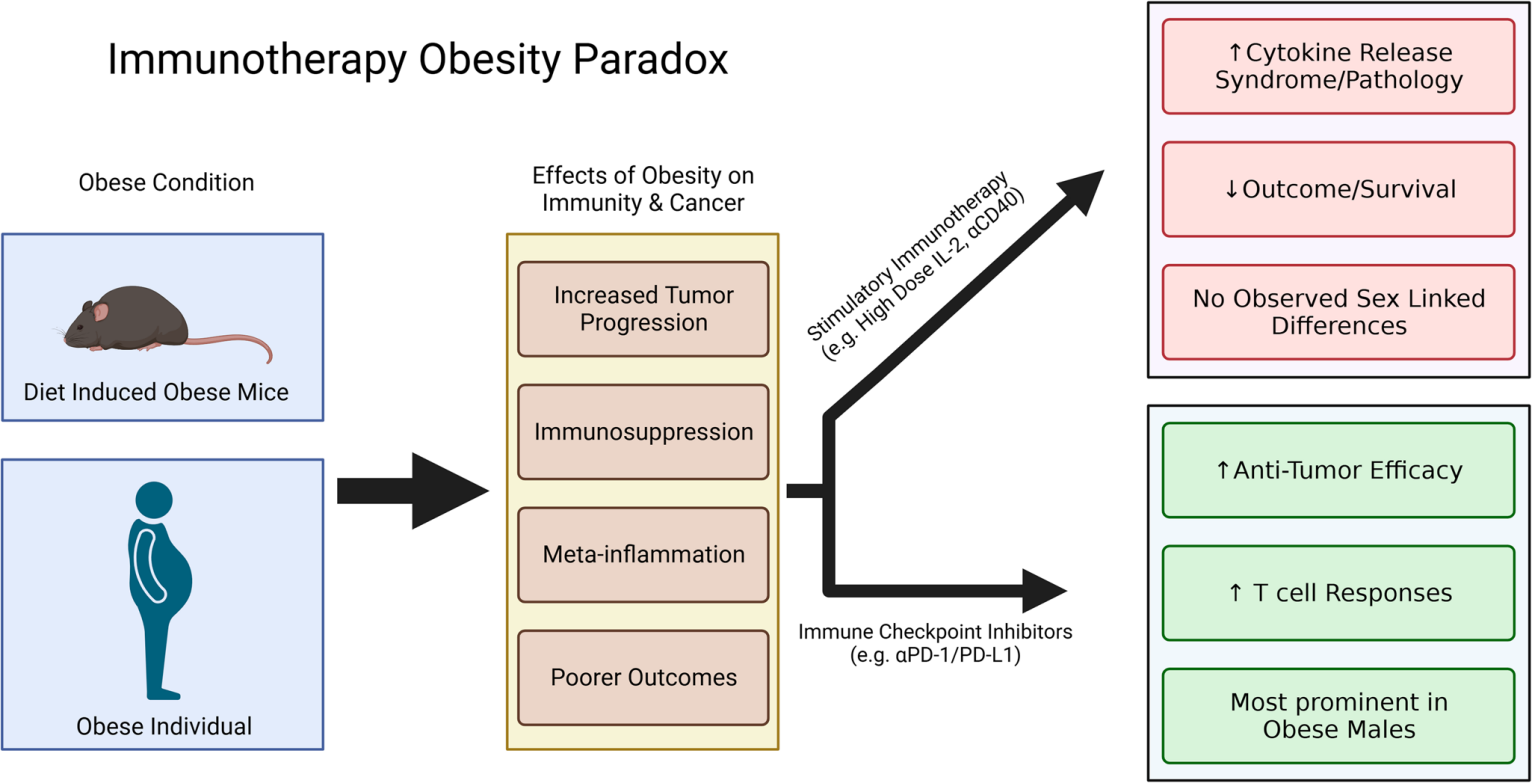
The immunotherapy obesity paradox. Obesity in patients and preclinical models is associated with several negative effects including increased tumor progression, immunosuppression, meta-inflammation, and overall poorer outcomes. In the inflammatory obese environment immunotherapy can have contrasting effects depending on whether they are immunostimulatory or targeted at releasing inhibitory mechanisms seen with immune checkpoint inhibitors in which increased anti-tumor efficacy is observed.

In recent years, obesity has gained considerable attention in the field of immunotherapy. Immunotherapy as a term is often too broadly applied and preclinical studies indicate there can be profound differences depending on whether it involves systemic immune stimulation versus Immune checkpoint inhibition (ICI). Regimens that involve strong systemic immune stimulation such as high dose cytokine therapies (e.g. IL-2) or allogeneic hematopoietic stem cell transplantations (allo-HSCT), have been associated with poorer outcomes due to increased inflammatory responses and multiorgan pathologies^13–15^. In contrast, ICI targeting the PD-1/PD-L1 axis was associated with greater antitumor effects and improved clinical outcome (e.g. improved survival) in obese patients suggesting an “Obesity Paradox”^16,17^ (Fig. 1).

Although several subsequent studies have also associated obesity with improved survival in patients treated with ICI, these findings are not universal^18^, and there is still much debate on whether obesity is an appropriate indicator of response, particularly if defined using BMI. Additional studies have suggested that obesity with consideration of factors, such as ratio of lean body mass or presence of sarcopenia, could be better determinants in predicting therapy outcomes^19,20^. Regardless, the Obesity Paradox seems to apply only for certain types of immunotherapeutic regimens that appear to remove the immune cell “brake” as opposed to applying more “gasoline” to fuel immune responses potentially due to the already existing nutrient-rich environment and meta-inflammatory state.

Interestingly, differences in response to ICI, and other cancer therapeutic regimens have been observed between obese male and female patients, McQuade et al.^16^ were the first to observe that obese male patients demonstrated the greatest benefit of ICI when compared to non-obese counterparts in the context of melanoma^16^. While other studies assessing melanoma^21,22^ have observed similar finding a recent meta-analysis of patients receiving ICI across several cancer types has further suggested that body composition associated survival may have a sex specific linkage^23^. However, other reports have contrasted with these observations reporting obesity’s association with ICI response is independent of patient sex^24^. These studies underscore how obesity may affect outcomes in male and female patients, an area of study that, in previous eras, has been neglected, and requires further investigation to assess whether this phenomenon is cancer specific, or linked to other biologic factors.

## Modeling obesity and cancer: using preclinical models that reflect human paradigms

Preclinical modeling is critical in allowing the dissection of the various factors affected by obesity and immune responses to cancer. Laboratory inbred mice housed under specific pathogen free conditions are the foundation of preclinical cancer modeling and allow for interrogation of the underlying biology of variables including obesity and sex under controlled situations. For the study of obesity in mice, there are two predominant strategies implemented either the use of monogenic mice dispositioned to develop obesity or polygenic diet induced obesity (DIO) models. Monogenic models utilize mice that are genetically deficient in key genes (e.g. mutated or lost) notably leptin (ob/ob mice) and leptin receptor (db/db mice) which each results in rapidly increased weight gain and metabolic deficiencies^25,26^. DIO models are generated by placing mice on a given diet, notably a high fat diet (HFD) or a Western diet (characterized by high fat and high sugar), over an extended period of time, to induce weight gain and an overt obese phenotype. DIO models have gained traction, given their greater applicability to human obesity and lack of genetic abnormalities^27^. A notable limitation of these murine models is the lack of an equivalent metric to BMI, with mouse obesity often being characterized by a weight differential from controls, which in some models or strains of mice can be variable and, at times, arbitrary. There is a real need to correlate weight gain with metabolic signature differences in preclinical models to better extrapolate to clinical data.

The use of syngeneic tumor transplantation models in DIO mice has already demonstrated applicability to human paradigms as seen in Wang et al.^17^, which demonstrated that tumor bearing DIO mice treated with anti-PD-1 received greater anti-tumor effects when compared to mice on control diets. This was observed even though tumors in DIO recipients grew at faster rates than controls and demonstrated an ability for anti-PD-1 treatment to both reduce tumor burden and improve survival in a preclinical model of oebsity^17^. A key limitation of using preclinical murine tumor models (subcutaneous) is the speed at which these tumors grow and the length of studies, which are not necessarily reflective of human cancers. Additionally, due to guidelines for humane endpoints in preclinical tumor studies readouts are often limited to tumor growth or a survival metric based upon approved endpoints (e.g. weight loss, tumor burden, body condition score, etc.) rather than a “true” survival. However, immunotherapy studies using DIO and lean mice in which tumors spontaneously arise, such as genetically engineered mouse tumor models (GEMM), are needed as they better mirror the clinical cancer scenario and better recapitulate the effects of obesity on tumor formation/progression, altered metabolic profile, and how these affect immune cells both in the tumor and peripherally.

The majority of preclinical studies evaluating the role of obesity on immunotherapy are performed using a single sex, even though evidence suggests that female mice are protected against HFD induced metabolic syndrome^28^, and are confined to a single mouse strain, more commonly C57BL//6 mice as other strains such as BALB/c are considered “resistant” to DIO phenotypes^29^. This is despite studies identifying sex as a significant modifier of the impact of HFD^28,30^. While both male and female mice can attain a similar obese phenotype from exposure to HFD overtime, female mice gain weight less rapidly, but then experience increased adiposity, and decreased lean muscle mass as they age, as well as differences in where fat is accumulated (visceral versus subcutaneous deposition)^31^. Obesity itself can also have a profound impact on sex hormone (including both androgens and estrogens) production^32^. The age of mice used is another key factor as most studies use young (8-12 weeks of age) mice. Even in models in which mice are placed on diet for several months this is analogous to a human age equivalent of 20-30 years old, whereas most cancer diagnoses are in individuals over the age of 55. This age discrepancy between the models likely has significant differential effects on immune status and obesity can further affect it particularly given that aging itself is also associated with a pro-inflammatory state. Further, the interaction between age and sex cannot be understated as there are substantive differences between mice and humans. Notably, female mice to not undergo menopause while humans do which should be considered when interpreting models intended to reflect older often postmenopausal human populations. In some circumstances use of ovariectomy has been used in murine models as a tool to better capture decreases in estrogens with age and has been further reviewed elsewhere^33^.

## Obesity: adiposity, inflammation and biomarkers

Obesity is a condition of excess body fat or adipose tissue (AT) and is often associated with a state of chronic inflammation which contributes to alterations in many metabolic and immune processes. AT is responsible for secreting a vast array of proteins, including hormones and cytokines, referred to as adipokines, which regulate a range of processes including appetite control, glucose homeostasis, insulin regulation, inflammation, tissue repair, and immune cell recruitment^34^. The inflammatory state observed in obesity stems from the stress imposed on adipocytes through the processes of hypertrophy (increase in adipocyte size) and hyperplasia (increase in adipocyte number), which occurs during excess accumulation of AT, notably white adipose tissue (WAT). The accumulation of WAT is a normal process of storing energy, often as triglycerides, which are then hydrolyzed during the generation of fatty acids for use by other organs during periods of energy deprivation^35^. However, obesity promotes adipokine dysregulation, release of free fatty acids through lipolysis, and secretion of inflammatory mediators; this altered state can promote apoptosis of adipocytes through hypertrophic expansion exacerbating inflammation, recruitment of immune cells, and metabolic alterations^36^.

Understanding the relationship between obesity and other ICI biomarkers may offer insight into what is driving the “obesity paradox”. A number of criteria have been identified as biomarkers for predicting response to ICI including PD-L1 expression^37–39^, tumor mutational burden (TMB)^39–41^, and microsatellite instability (MSI) or DNA mismatch repair (dMMR)^42–44^. WAT has been identified as a key reservoir of PD-L1^45^ and notably, has been reported to be overexpressed in the WAT of DIO mice accompanied by increased PD-1 in VAT but not SAT^46^. These data suggest a link between obesity and PD-L1 which other studies have supported through indications that PD-L1 plays a direct role in mediating AT inflammation and may be upregulated as a counter measure to obesity induced inflammation^47,48^. Further connection PD-L1 is seen in preclinical studies where inflammatory mediators secreted by adipocytes have been implicated in upregulated expression of PD-L1 on cancer cells^49^. TMB is a measure of mutations per megabase (Mb) for a tumor and a predictive biomarker for ICI success, yet there seems to be little evidence linking obesity and TMB. Hahn et al.^50^ reported no difference in TMB between overweight and obese melanoma patients when compared to patients with BMI < 25; they also identified that BMI was not associated with DNA mutations or copy number variations^50^. Similarly, Sanchez et al.^51^ in a study evaluating patients with renal cell carcinoma (RCC) observed no difference in TMB between obese and non-obese patients^51^. MSI/dMMR particularly in the context of colorectal cancers have been predictive of ICI response. However, studies have indicated that colon cancers from obese patients are less likely to display dMMR^52^ and although obesity is linked with increased risk of colorectal cancer high BMI has been associated with MS-stable or MSI- low tumor phenotypes^53^. Further investigation is warranted to elucidate how obesity may relate to these and other biomarkers and may help in interrogating why in some instances obesity is associated with improved ICI response.

## The immune landscape in obesity and the tumor microenvironment (TME)

AT is composed of a diverse variety of cell types including adipocytes, endothelial cells, fibroblasts, and many immune cell types each of which can be significantly affected by excess adiposity. Obesity can mediate immune modulation within AT, visceral organs, and tumors. Macrophages are the most abundant immune cells in AT, and under obese conditions they further localize increasing in number as AT expands^54^. In addition to increased infiltration, obesity also promotes the local proliferation of AT macrophages^55^. The influx of macrophages into the AT contributes to obesity induced inflammation through secretion of additional inflammatory mediators including IL-6 and TNF^54,56^. Macrophages can play a prominent role in the immune polarization of AT and tumors, being broadly delineated into either a “classical” pro-inflammatory M1 or an anti-inflammatory M2 phenotype.

Using a DIO model implementing a HFD Lumeng et al.^57^ observed that obesity can significantly alter macrophage polarization within AT shifting from an M2 phenotype observed in lean control mice to an M1 inflammatory phenotype indicated by increased expression of TNF and iNOS^57^. Although obesity has been associated with an increased M1 presence in AT, studies from DIO models have indicated that within the tumor microenvironment (TME), obesity can promote M2 skewing that adversely impacts immune responses and promotes tumor growth^58,59^. It is still not clear whether metabolic perturbations of obesity are responsible for this skewing, or the meta-inflammatory state associated with obesity.

Contributing to immunosuppressive macrophage polarization and immunosuppression in general, are myeloid derived suppressor cells (MDSCs), which have been observed to be elevated in obesity in part due to increased IL-6 and other pro-inflammatory cytokines. MDSCs act as a counterbalance to metabolic dysfunction and adipose tissue inflammation, consequently suppressing CD8 T cells and promoting M2 polarization^60^. MDSCs have been associated with tumor progression and immune suppression. Gibson et al.^61^ demonstrated this by observing, in a breast cancer model, that DIO mice demonstrated increased immunosuppression through the increased recruitment of MDSCs to the TME. These MDSCs promoted apoptosis of CD8 T cells and mediated resistance to stimulatory CpG immunotherapy^61^. Additional studies have corroborated elevated MDSCs in the TME using DIO tumor models, observing increased immune suppressive effects in groups receiving HFD, while also presenting evidence suggesting MDSCs contribute to protection from metabolic dysfunction^62,63^.

T-cells are the second most abundant immune cell in AT and their consideration is of particular importance when discussing cancer, ICI, and ICI efficacy in obesity. Obesity has been implicated in either suppressing or impairing a number of T cell functions, notably restricting T cell receptor (TCR) repertoire^64^, reducing TCR sensitivity, and promoting T cell exhaustion^65^. Using a DIO model of autoimmunity, obesity was identified as impairing T cell priming, yet was restored through use of PD-1 blockade^66^. Dyck et al.^67^ using syngeneic colorectal and melanoma tumor models illustrate the suppressive effects of HFD and obesity on CD8 + T cells that anti-PD-1 treatment could reverse^57^. Although, the investigators identify no significant differences in glycolysis or OXPHOS pathways between CD8 + T cells from HFD and control mice they did observe decreased levels of local kynurenine which they use as an indirect surrogate for cell activity. They also observed decreased levels of glutamine, arginine, ornithine, and kynurenine in the serum of mice fed HFD^67^.

While CD8 T cells have been identified as the predominant immune cell affected by obesity responsible for anti-tumor effects T regulatory cells (Tregs) have also been implicated as cells influenced by obesity with some studies demonstrating increased numbers of Tregs while others observing decreased numbers, notably in the AT^68^. Decreased levels of Tregs in obesity in adipose tissue has been suggested to contribute to inflammatory escalation and insulin resistance^69,70^. This is further supported by clinical data suggesting that Tregs are reduced in the circulation of obese individuals when compared to nonobese counterparts^71^. The inflammatory state of obesity has also been observed to promote an imbalance in CD4 T cells, in which Tregs decline as proinflammatory T helper 1 (Th1) and T helper 17 (Th17) cells expand^72^. However, preclinical data has suggested there are sex differences in Tregs, with estrogen mediating localization of Tregs to AT^73^. The overall effects on T cells appears to be suppressive and proinflammatory.

## Obesity induced metabolic effects on cancer and immune cells

Underpinning the association between obesity with cancer incidence and progression are alterations in metabolism both in tumors and immune cells. Hahn et al.^50^ investigated how obesity was altering the TME in samples from 782 patients with metastatic melanoma grouped from several cohorts in which they assessed DNA mutations, gene set enrichment analysis, microbiome and metabolic profiling based on high (≥25) and low (<25) BMI. The study observed no differences in DNA mutations or differences in diversity or taxonomy in microbiome between high and low BMI groups, but the study did identify distinct differences in the metabolic profile in which tumors from high BMI patients were surprisingly more quiescent characterized by downregulated glycolysis and OXPHOS pathways^50^. Further, metabolite analysis revealed significant differences in citrate and succinate both of which were decreased in high BMI patients suggesting potential alterations in the TCA cycle^50^. This quiescent phenotype in the clinical samples would appear to be at variance with preclinical studies demonstrating more rapid tumor progression^17,67,74^, but does fit in line with the concept that the increased tumor progression is mediated more by immune suppression rather than increased nutrients on the tumor itself^67,74^. Given the heterogeneity of human cancer cells within the tumor, more needs to be assessed on whether the effects of obesity on cancer proliferation may be time or stage-dependent or in different regions of the tumor given that hypoxia is also associated with progression^75^.

Obesity is associated with increased free fatty acids (FFA), which in turn have been associated with increased cancer progression^76^. Altered fatty acid metabolism has been identified as a potential mechanism of cancer progression but has also been linked to multiple effects on immune cell metabolism which may vary depending on the particular immune cell-type. For example, obesity has been associated with deficits in both mouse and human NK cell numbers and function which has also been linked directly to metabolites in the obese state^77–79^. An example of this is illustrated by Michelet et al.^78^ in which they demonstrate in a preclinical murine model that Natural Killer (NK) cells upregulate lipid metabolism in response to HFD and correspondingly downregulate cytotoxicity, becoming impaired^78^. They further connect NK cell dysfunction with decreased glycolysis and OXPHOS pathways as well as impairment of mechanistic target of rapamycin complex 1 (mTORC1) and identified lipids, such as palmitate and oleate, that could induce this dysfunction. They also observed that this NK cell dysfunction induced through lipid exposure/treatment could be reversed^78^. Another study by Jiao et al.^79^ also observed functional defects in NK cells during cases of obesity and linked these defects with increased lipid exposure, yet identified that exposure acted through suppression of c-Myc acetylation ultimately impeding NK cell effector function^79^. Thus, in the context of NK cells the metabolic effects of obesity appear tightly linked with FFAs and lipids, which can mediate NK cell functional impairment. As discussed previously, the role of diet (rather than only HFD) needs to be better explored as well as in the context of aging.

Ringel et al.^74^ also evaluated the role of obesity on immune metabolism looking in the TME. They utilized mice on a HFD and challenged with several syngeneic tumor lines, highlighting increased tumor growth in mice exposed to HFD and through use of T cell depletion, and subsequent sequencing analysis elucidated that this observed tumor-promoting effect was immune mediated and facilitated by metabolic remodeling, notably within CD8 + T cells^74^. The authors noted that obesity alters the TME observing that glycolytic markers decreased in tumor cells accompanied by promotion of fatty acid oxidation, while this did not occur in tumor infiltrating CD8 + T cells^74^. These data suggest that tumor and immune cells adapt differently in their competition for resources and nutrients in the obese TME. Though informative many preclinical studies center on assessment of particular immune cell-types in which the metabolic effects may differ considerably and may be cancer-specific.

## The obesity paradox and immune checkpoint inhibitors (ICI)

As previously noted, obesity has paradoxically been associated with more efficacious clinical responses with ICIs^16,17,21,51,80–89^. Interrogating the role of obesity in clinical outcomes, as related to ICI, has been challenging, as patient populations are genetically and demographically diverse, with different clinical histories and cancer types/treatments. However, multiple cancer types have correlated obesity, as measured by BMI, with more favorable responses to ICI, including increased progression free and overall survival^17,86^. However, although there is mounting evidence supporting the association of obesity and improved outcomes with ICI, there are conflicting interpretations on whether there truly is an obesity paradox, and whether there are additional parameters that should be considered which may allow for greater delineation of protective effects^18,51,90,91^.

Some cancers, like non-small cell lung cancer (NSCLC) and melanoma, have been shown by multiple studies to promote the obesity paradox^16,17,21,82,84,92–95^. Other cancers, such as renal cell carcinoma (RCC), have had several studies conducted indicating that obesity is not associated with improved survival, predictive of ICI response, or is even associated with poorer outcome^51,90,91^. However, a study by Lalani et al. (2021) and a meta-analysis conducted by Takemura et al.^87^ do conclude that BMI plays a significant prognostic role in RCC^87^. These data together do suggest that cancer type may play a major role in the prognostic value of obesity.

BMI is a standardized and easily applied metric to diagnose obesity, however the inability to discern between lean and fat body mass and low sensitivity suggest that BMI may be underdiagnosing those that are actually obese^7,96^. Even when evaluating studies using BMI as a measure of obesity, interpretation can be made difficult by studies implementing different cut-offs on criteria used. In some studies, a broad definition has been applied assessing both overweight and obese populations together, utilizing a BMI > 25, while other analyses have considered only those with BMI > 30^22,82^. Finally, BMI scores of even greater than 40 and up are being observed and this further complicates interpretation given that morbidly obese individuals also present with co-morbidities^21,92^.

Additional studies have identified nuance in the BMI obesity paradox, observing that the association of BMI and survival is not always linear. These studies identify that increasing BMI appears to be beneficial, until a threshold, in which increases in BMI are no longer associated with improved ICI response^21,92^. Interestingly, ICI dose has corelated with outcome, where high BMI was associated with improved overall survival, yet this was utilizing weight-based dosing administration, rather than a fixed regimen^22^. It may be necessary to evaluate obesity in a more comprehensive manner by using alternatives to BMI, such as Visceral Fat Index (VFI), which can drastically impact interpretation of evidence^97^. Furthermore, in a study evaluating the efficacy of ICI in metastatic melanoma, parameters including skeletal muscle index (SMI), VFI, and systemic inflammation index (SII) were used in place of BMI, and it was observed that both VFI and SII were predictive of ICI success^98^.

A number of studies have also identified that beyond the presence of excess AT the type and location of adipose tissue present can be predictive of ICI success though the results between studies is varied^99–101^. A small retrospective study from Martini et al.^100^ evaluating patients with melanoma, gastrointestinal, lung, breast, and head and neck cancers treated with immunotherapy identified that increased BMI was associated with prolonged patient survival^100^. They also identified that indexes for subcutaneous fat (SFI) and intramuscular fat (IFI) were also predictive of response with increased SFI and decreased IFI each being associated with improved survival in immunotherapy treated patients^100^. Contrastingly, a retrospective study from Mengoni et al.^99^ of melanoma patients undergoing ICI identified that decreased subcutaneous adipose tissue, which the authors assess as subcutaneous adipose tissue gauge index (SATGI), is associated with improved progression free survival (PFS) and a predictive biomarker for ICI response^99^. Another study evaluating patients with advanced stage melanoma, gastrointestinal, lung and other cancers that were treated with ICIs, although observing no significant association of BMI on PFS, did observe that values including subcutaneous fat area (SFA) and visceral fat area (VFA) were predictive of increased OS in responders with higher VFA/SFA ratios being associated with better outcomes^101^. These data suggest that not only is there an association between excess adiposity and ICI responses, but that adipose tissue type and distribution play a role as well. The implications of AT distribution also impact the interpretation of the role patient sex on ICI outcome given male patients are disposed toward increased VAT while female patients ae disposed toward SAT^102^.

While obesity and AT has paradoxically been identified as an indicator of improved outcome to ICI, increased lean muscle mass has also been associated with improved outcome while decreased muscle density has been implicated with decreased survival^103^. Decazes et al.^104^ demonstrated this by utilizing 3D CT scans to evaluate both subcutaneous fat mass (SFM) and muscle body mass (MBM) in a population of 623 melanoma and NSCLC patients treated with ICI^104^. They observed that MBM was able to predict ICI response in both melanoma and NSCLC, but interestingly MBM and SFM taken together demonstrated a complementary prognostic value allowing for stratification of patients on both criteria with low SFM and MBM patients having the poorest prognosis^104^. This follows data from Takenaka et al.^105^, who performed a pan-tumor meta-analysis and identified sarcopenia as an indicator of poor prognosis to ICI. Similarly, Chen et al.^106^, in a retrospective analysis performed on 138 patients treated for advanced hepatocellular carcinoma, revealed sarcopenia was associated with significantly poorer survival (PFS and OS) and that patients with sarcopenic obesity also demonstrated significantly poorer survival^105,106^. Importantly, when considering muscle mass, both age and sex are relevant parameters, given muscle mass steadily declines with age and females tend to have lower muscle mass when compared to males.

## The role of sex-linked differences on the obesity paradox

Patient sex is a lesser studied variable that has been associated with distinct differences in immune responses between male and female patients as well as cancer treatment outcome (including ICI)^107^ and may be a key factor in influencing the obesity paradox. Evidence supporting the link between patient sex, obesity status, and ICI have been indicated by several studies, most notably in cases of melanoma but also in other cancers including urothelial carcinoma, in which significant gains in survival have been observed in male obese patients receiving ICI, while less so in obese female patients^16,21,22,108^.

The observation that sex-linked factors could affect immunotherapy and obesity was highlighted by McQuade et al.^16^ in a retrospective study of 2046 patients with metastatic melanoma, in which the effects of obesity (defined using BMI) on patient outcome was evaluated across several treatments, including chemotherapy, targeted therapy and immunotherapy. The study observed that obesity had no significant association with chemotherapy outcomes, yet obesity was associated with improved survival for both targeted therapy and immunotherapy. However, patient sex was identified as a key determinant of outcome, with obese males having significant increases in survival, whereas these protective effects were not observed in obese female patients^16^. An important caveat with these data is that while there are not significant increases in the median PFS or OS of overweight/obese female patients this is in part due to non-overweight/obese female patients having increased PFS and OS than male counterparts. This may suggest that immunotherapy in the context of obesity may be equalizing differences in survival between male and female patients.

Naik et al.^21^ performed a meta-analysis assessing melanoma patients receiving ICI therapy between 2014 and 2016 and assessed the impact of obesity. The study observed that overall, overweigh/obese patients did have a significant advantage in progression free and overall survival, as compared to their normal weight counterparts. Importantly, similar to McQuade, they identified that there was an impact of sex on survival outcomes, with improved survival being demonstrated predominately by overweight/obese males^21^. Adding to this observation, they assessed serum creatinine levels, which can be used as a surrogate for skeletal muscle mass and observed that low creatine levels were associated with an attenuated impact of obesity on improved survival in male and female patients. Notably, females were also identified as having lower levels of creatine generally suggesting that muscle mass may be a sex liked parameter to consider in the context of obesity^21^.

A study from Trinkner et al.^23^ performed a systematic literature search and meta-analysis, including studies performed from 2017-2022 across multiple tumor types, to evaluate the influence of body composition and obesity on ICI therapy outcomes. From their analysis, they identified that overweight and obese patients, based on WHO BMI classifications, had significant progression free and overall survival benefits, when compared to normal weight counterparts. Supporting data generated by others, subgroup analysis revealed that male overweight or obese patients demonstrated benefit, with no difference observed with overweight or obese female patients^23^.

Huang et al.^108^ present a retrospective analysis of 215 patients with metastatic urothelial carcinoma and treated with ICI therapy in which they set BMI criteria as greater than or equal to 25 or less than 25. This study identifies that a BMI ≥ 25 is an independent factor for predicting OS in patients treated with ICI and further found that male patients from the BMI ≥ 25 group observed greater benefits from ICI treatment^108^.

Though studies do point to differential outcomes of obese patients based on sex, this has not been found with all studies and has been predominately in cases of melanoma. This is an important caveat given Jang et al.^109^ observed that in cases of advanced melanoma female patients may not receive the same benefit as males in a treatment regimen of combination nivolumab and ipilimumab, though they did not stratify on BMI^109^. Further, studies through a pooled analysis of 4090 patients have identified an association between obesity and ICI response, yet found this association independent of patient sex^24^. In totality, these data suggest that while obesity has meaningful implications for the outcomes of patients treated with ICI, obesity does not work in isolation and should be considered in tandem with variables such as patient cancer type, sex and age, which may offer deeper insights into underlying mechanisms.

## The role of sex hormones on immunotherapy

Data demonstrating that patient sex correlates to ICI outcomes in the context of obesity provides an opportunity to unravel features that may be important for effective immunotherapy treatment. Sex derived differences often originate from two key aspects either genomic difference based on X and Y chromosomes or differences in sex hormones including estrogens and androgens. Obesity has been observed to have distinct dimorphic effects based on sex notably the distribution of AT and changes in the secretion of sex hormones^110^. In the context of immunotherapy, obesity and sex may be crucial prognostic indicators due to these distinctions and alterations in body mass composition, immune phenotype and metabolic effects.

Sex hormones are signaling steroids that interact with hormone receptors eliciting a wide range of responses notably in reproductive processes, but they can influence both metabolism and immunity. Androgens, estrogens, and progestogens are the three broad classes of sex hormones with testosterone and estradiol being two of the most notable examples of sex hormones. Testosterone is often most prominent in males, while estrogen is typically more abundant in females though both hormones are present regardless of sex^111^. The kinetics of sex hormones overtime is variable, but when measured in serum can be described by increases during puberty followed by a steady decline with age with a notable sharp decline in estrogens for females around fifty years in age^111^. As previously noted modeling the kinetics of sex hormones preclinically can be challenging due to physiologic differences notably that female mice do not undergo menopause. Additional considerations should be taken when comparing hormones from female mice in preclinical models as hormones can vary depending on the estrous cycle which can be characterized estradiol steadily increasing followed by a decrease, which takes place over the course of a four to five day cycle^112^.

Sex hormone levels can be significantly altered in obesity having contrasting effects in men and women. As a site of estrogen synthesis increased AT can result in elevated estrogen levels^113^. Males with obesity have been associated with lowered testosterone and also elevated levels of estrogen^114,115^. In females, sex hormones in obesity can be quite variable given estrogens traditionally decline following menopause and estrogen production often shifts to AT, which is in excess during obesity. The distribution of AT is a prominent difference in obese males and females, with obese females generally having greater subcutaneous AT (SAT) and decreased visceral AT (VAT), when compared to males^116^. AT distribution can have meaningful implications, with excess VAT being associated with morbidity and mortality more so than SAT^102^. The characterization of AT can also be impactful as a major reservoir for immune cells, including memory T cells^117^. As previously discussed, these differences in adipose tissue distribution may have meaningful implications for ICI treatment outcome^70,98–100^. When evaluating cancer and obesity, transcriptionally, obesity promotes metabolic dysregulation within the tumor microenvironment, which is observed in both male and female patients, yet the latter demonstrate more prominent effects^118^.

Both estrogens and androgen, have been reported to mediate a number of effects on both cancer and the efficacy of immunotherapy^119,120^. Increased levels of estrogen have been associated with increased Tregs and elevated PD-1 expression on T cells, while low levels of estrogen have been observed to promote proinflammatory Th1 differentiation^73,121^. Androgens have generally been implicated as immunosuppressive affecting both innate and adaptive immune compartments^122^. The net effect of sex hormones is challenging to assess, as there are contrasting paradigms describing whether a particular hormonal signaling pathway is beneficial or detrimental, and which types of immune cells are most impacted.

Chakraborty et al.^123^ observed through CIBERSORT immune deconvolution of bulk RNA-sequencing from melanoma ICI responders and non-responders, that polarization/functionality of intertumoral macrophages was associated with ICI response^123^. They interrogated this observation preclinically using murine melanoma models and demonstrated that estrogen accelerates tumor growth and that this was due, at least, in part to M2 macrophage polarization affecting CD8 T cell effector function. They further demonstrated that anti-tumor efficacy with anti-PD-1 could be improved, indicated by reduced tumor burden, through inhibition of estrogen signaling, which was demonstrated by estrogen receptor deletion on macrophages or use of fulvestrant to block estrogen signaling^123^.

Conversely, androgens have been implicated in the poor response of ICI. Guan et al.^124^ demonstrated in a prostate cancer model that the use of androgen receptor blockade improved responsiveness to ICI targeting the PD-1/L1 axis through direct interaction with CD8 T cells, improving effector function^124^. Yang et al.^64^ similarly reported that androgen receptor signaling can suppress antitumor responses through direct interaction with CD8 T cells, identifying that androgen receptor signaling disrupts the preservation of stem cell like CD8 T cells and is correlated with CD8 T cell exhaustion in instances of human cancer^125^. The use of either surgical castration or small molecule inhibitors in male mice has been observed to reduce testosterone synthesis and to also enhance the antitumor activity of T cells^126^.

**Fig. 2:**
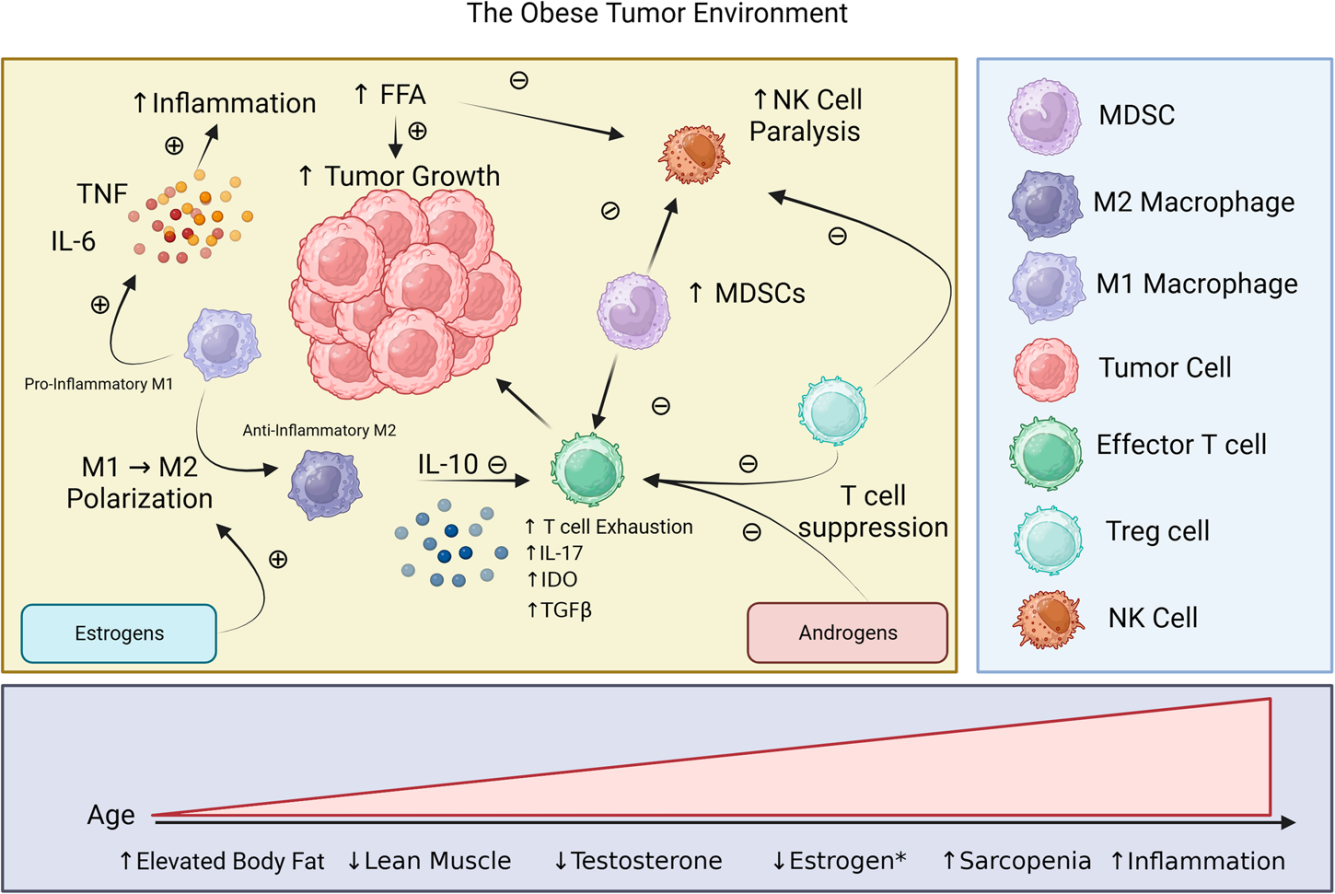
The obese tumor environment. Within the tumor microenvironment (TME) obesity promotes immunosuppression through macrophage polarization (M1→ M2), T cell exhaustion, and suppression of effector cells myeloid derived suppressor cells (MDSCs) and T regulatory cells (Treg), which can be further augmented by sex hormones promoting further suppression or polarization.*Estrogen levels vary between individuals, production from ovaries declines post menopause, production in adipose tissue can increase and become a significant source of estrogen production post menopause.

These data suggest that both estrogen and androgen signaling directly influence immune cells in different ways, indicating that there is not necessarily one hormonal signaling pathway that is beneficial to anti-tumor responses and one that isn’t (Fig. 2). Particularly in obesity, in which both men and women experience modulation of hormonal levels, it poses a question on whether there is an “ideal” balance of hormonal signaling that promotes an effective response. Further, these questions of the effects of hormones are not in a vacuum and occur in the context of differences in AT distribution and differences in lean body mass. Questions on how age should be considered as parameter need to be considered, as lean muscle and sex hormone levels can decline with increasing age.

## Discussion and future research directions

Great attention has been given to the role of obesity in ICI therapy, yet many questions remain as to how the obese environment could be driving favorable outcomes or whether an obesity paradox exists at all. Care must be taken when making associations between obesity and ICI outcome, as there are limitations in modeling/studying, most notably a heavy reliance on BMI as a diagnostic tool and preclinical modeling that may not appropriately mirror human paradigms. Obesity has been linked with chronic inflammation, immune suppression, altered metabolism, and increased cancer incidence^12,27,74^. Additionally, obesity has been associated with increased T cell exhaustion and upregulation of inhibitory proteins including PD-1, LAG-3 and TIM-3^17,127^. Further, obesity promotes increased levels of PD-L1^49^ and MDSCs are elevated in the obese tumor environment adding to immune suppresion^62,63,128^. However, a number of studies have observed findings that support a surprising association of increased BMI with improved survival in patients treated with ICI^16,21,82–86^, though this is not universally observed^51,81,88,129^, including indications that in the context of obesity particular cancers like RCC may receive less benefit^90,91^. It has been hypothesized that exhausted T cells respond better to ICI and consequently the increased T cell dysfunction, in part driven by inhibitory proteins like PD-1, may result in T cells in the obese environment being more susceptible to treatment^17,130^. However, the true mechanisms underlying the obesity paradox remain unclear.

McQuade et al.^16^ made the observation that male obese melanoma patients received greater benefit from ICI than obese female counterparts^16^. Although, there are caveats in the data interpretation the improved response of obese males is informative suggesting contributions of sex linked factors including sex hormones, AT distribution, and amount of lean body mass each of which has been implicated in the efficacy of ICI^21,99,103,123^.

Sex hormone signaling impacts many pathways, both directly impacting cancer growth, metabolism, and the immune system^122,123,125^. These hormonal differences, coupled with obesity, result in unique immune and metabolic environments for male and female patients. In the context of ICI, studies have implicated that both the signaling of estrogens and androgens can have a negative impact on outcomes^123–125^, suggesting that hormone signaling may rely upon a balance of a hormone ratio, as opposed to, the presence of increases in a single signaling pathway. Yang et al.^64^ suggest that androgen signaling can directly suppress CD8 T cells, while Chakraborty et al.^123^ identify direct effects of estrogen signaling on macrophage polarization toward an M2 phenotype^123,125^. Taking these signaling pathways together obese males, which have been characterized by decreased testosterone and already have lower estrogen than female counterparts, may strike a balance for effective ICI therapy. This concept of identifying a hormonal balance that ideally suits ICI responses can have substantive implications and may shed light on why male obese patients generally respond better to ICI and whether hormonal assessment should be considered as a prognostic factor.

**Fig. 3:**
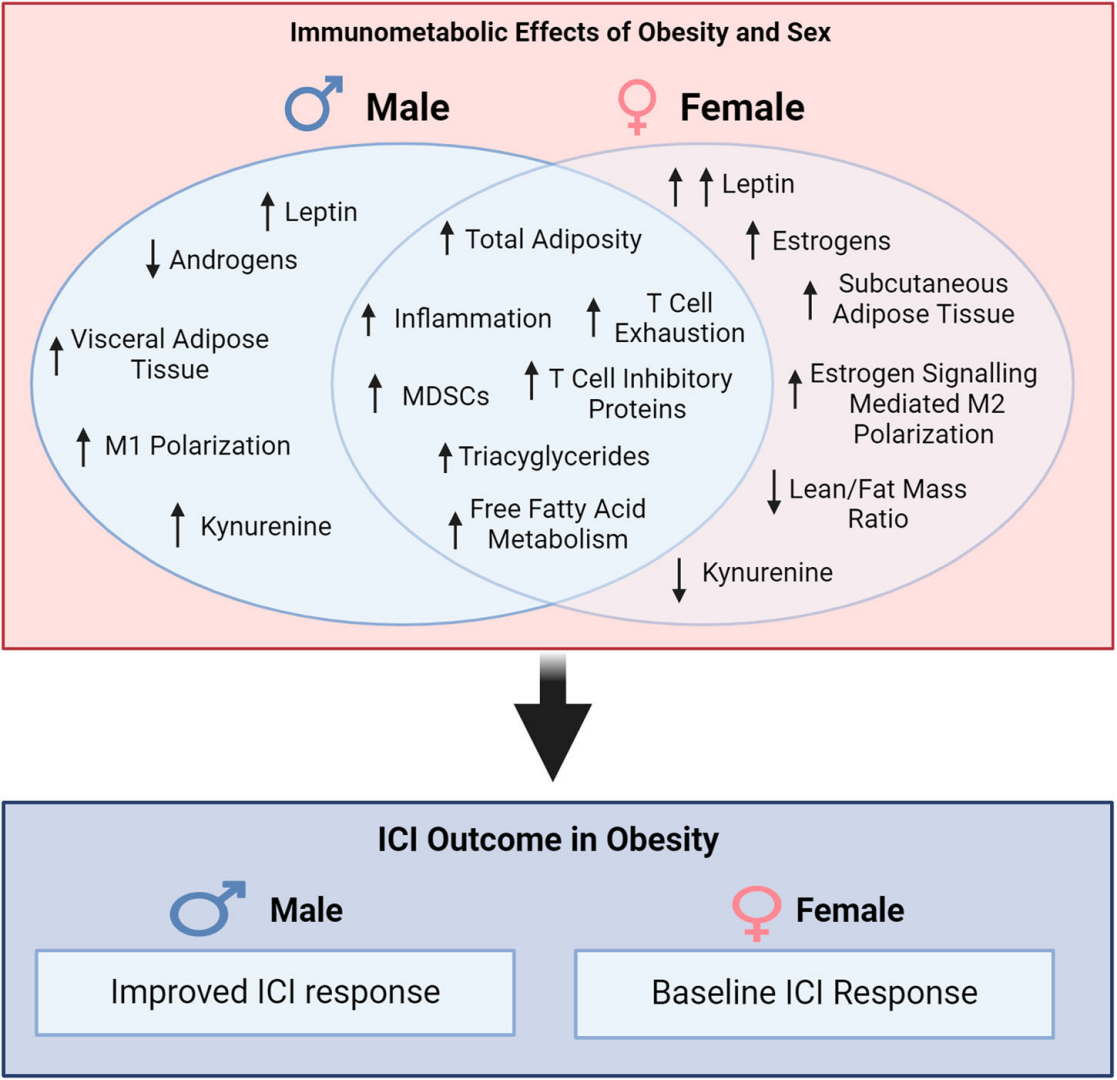
Immunometabolic effects of obesity and sex. Obesity elicits changes in physiology, metabolism, and immunity some of which are sexually dimorphic notably adipose tissue distribution, macrophage polarization, and metabolism. Obesity in males is depicted with decreased androgens, increased visceral adipose tissue, increased M1 macrophage polarization, and increased Kynurenine. Obesity in females’ id depicted as increased leptin levels when compared to males, elevated estrogen with adiposity, disposition toward subcutaneous adipose tissue, M2 polarization mediated through estrogen signaling, a decreased lean-to fat mass ratio, and decreases in Kynurenine. A hypothesized link of putative factors to ICI outcome is depicted with obese males benefitting greater than obese females when compared to respective nonobese/low BMI counterparts.

Studies are beginning to identify that both fat distribution and muscle mass are important prognostic indicators, with the proportion of an individual’s lean muscle mass being implicated as a factor linked to immunotherapy success^131^. Given sexual dimorphisms for AT distribution and muscle mass this may suggest a connection driving sex differences in the “obesity paradox”. It is unlikely that patient sex alone is the only variable to consider; preclinical studies have demonstrated that aging in male mice can modulate the effects of obesity impairing response to immunotherapy^127^. The type of fat (e.g. brown or white adipose tissue) may also play a role as each are distinctly affected by metabolic changes and sex hormones which has been reviewed elsewhere^132,133^. The totality of these factors including obesity, sex, lean muscle, and age, may be a contributing to distinct outcomes to ICI (Fig. 3) in addition to other variables such as diet and microbiome.

Regarding the clinical implications of these findings, the next generation of clinical trials could potentially investigate the combination of hormonal therapy targeting estrogen as well as metabolism-targeted treatments to enhance the clinical efficacy of ICIs. The interplay of obesity, gut microbiome, and diet in ICI therapy has also been explored, with potential implications for treatment effectiveness^134^. It will also be important to determine if other immune checkpoints like LAG-3 and TIGIT blockade on how sex and obesity may affect efficacy. In conclusion, the obesity paradox in cancer is closely interrelated with sex, which has been shown to have a an impact on ICI outcomes^16,21,23,108^. While the differences have largely been attributed to endocrine and metabolic changes, further research is required to understand the underlying mechanisms and to better develop effective, personalized cancer immunotherapies.
